# Comparison of sterilisation methods for deer velvet antler extracts and their impact on anticancer activity

**DOI:** 10.3389/fphar.2026.1782990

**Published:** 2026-04-20

**Authors:** Nicolás Alegría-Aravena, Josefa Quiroz-Troncoso, Marta Sánchez-Díez, Clara Gavira-O'Neill, Raquel González-Martos, Irene Arroyo-González, Datao Wang, Carlos de Cabo, Andrés J. García-Díaz, María-Pilar López-Garrido, Martina Pérez Serrano, Francisco Sánchez-Sánchez, Tomás Landete-Castillejos, Louis Chonco, Carmen Ramírez-Castillejo

**Affiliations:** 1 IDR, IREC and ETSIAMB, University of Castilla-La Mancha (UCLM), Albacete, Spain; 2 Asociación Española Contra el Cáncer (AECC)-Fundación Científica AECC, Albacete, Spain; 3 Institute for Health Research of Castilla-La Mancha (IDISCAM), Albacete, Spain; 4 Centro de Tecnología Biomédica (CTB), ETSIAAB, Universidad Politécnica de Madrid, Madrid, Spain; 5 Department of Oncology, Instituto de Investigación Sanitaria San Carlos (IdISSC), Madrid, Spain; 6 Institute of Special Animal and Plant Sciences, Chinese Academy of Agricultural Science (CAAS), Changchun, China; 7 Research Department, Neuropsychopharmacology Unit, Complejo Hospitalario Universitario de Albacete (CHUA), Albacete, Spain; 8 Laboratorio de Genética Médica, Instituto de Biomedicina (IB), Universidad de Castilla-La Mancha (UCLM), Albacete, Spain; 9 Departamento de Producción Agraria, ETSIAAB, Universidad Politécnica de Madrid, Madrid, Spain

**Keywords:** anticancer activity, colorectal cancer, deer antler extract, deer velvet antler, natural bioactive compounds, sterilisation methods

## Abstract

Natural extracts tested for potential effects in human health are susceptible to contamination, particularly when samples are often obtained from outdoor environments. Deer velvet antler (DVA) extracts have broad anticancer effects. As sterile samples are required for effective experimentation in cell cultures, we evaluated the most common sterilisation methods to get rid of microbial contamination. We also investigated whether the sterilisation method affected the anti-cancer activity of the DVA extract on human tumour cells. Two antler sections (tip and base) were subjected to water-based extraction. This study compares lyophilised and non-lyophilised DVA extracts. Subsequently, DMEM and LB culture media containing DVA extract were assessed for contamination. The amount of protein was quantified by BCA and visualised by polyacrylamide gel electrophoresis. To analyse the anticancer effect, a cell viability assay was performed on colorectal cancer cell lines. Finally, tumour biomarkers were evaluated by flow cytometry in colorectal cancer cells. Filtration was the sterilisation method that removed the highest microbial load. It reduced cell viability at a concentration of 1 mg/mL of total protein by up to 37% ± 10% (DVA-T) and by up to 69% ± 8% (DVA-B). Furthermore, the protein expression levels of SW480 colorectal cancer cells exhibited a significant increase in response to lyophilised DVA extracts in comparison to non-lyophilised extracts. The data obtained from this study indicate that the selected sterilization approaches allow preservation of protein integrity and *in vitro* bioactivity of DVA extracts, supporting their standardized preparation for further biological evaluation.

## 1 Introduction

Deer antlers are unique among mammals because they are cast and grow every year, making them the only case of complete regeneration in mammals (Landete-Castillejos et al., 2019). The growth effort is outstanding, with red deer reaching a weight of more than 15 kg and a length of 120 cm in about 3.5 months from spring to summer (Gaspar-López et al., 2010; Gomez et al., 2013). This represents an astonishing growth rate in the tips (where they grow) of up to 2.75 cm/d in length and more than 20 cm^2^/d of skin (Landete-Castillejos et al., 2019). This rapid growth involves the presence of very active molecules which can confer potential applications in medicine, the most prominent of which is the anticancer activity of the growing antler extract (deer velvet antler or DVA (Wang and Landete-Castillejos, 2023)).

Antlers regrow annually owing to very fast cell proliferation that surpasses even cancerous tissue growth (Goss, 1983). Wang et al. (2019) showed that this is due to the high expression of proto-oncogene, although the effect of antitumor mechanisms to avoid the ristk of proto-oncogene expression leading to cancer, called Pardal effect, is applicable to all fast stem-cell proliferation and not just for antler growth (Pardal et al., 2005). Back to deer antlers, the study mentioned found that gene expression profiles were more highly correlated between antlers and human osteosarcoma (r = 0.67–0.78) than between antlers and human normal growth bone tissue (r = 0.33–0.47), a fact later confirmed and extended recently (Ba et al., 2025). As a result, the study postulated that deer have evolved several tumour suppressor genes (e.g., TP53) to control the high risk of developing cancer. Ba et al. (2025) showed that the tumour suppressor effect is based mainly in inducing high levels of apoptosis rather than DNA repair mechanisms. In both cases, this may explain why several studies have found *in vitro* and *in vivo* anticancer effects of DVA in human tumours such as glioblastoma (GBM; Chonco et al., 2021), prostate (Yang et al., 2017; Tang et al., 2019) and hormone-sensitive cancers such as breast (Li et al., 2022). In addition to these human tumours growing in mice, DVA has also shown anticancer effects by promoting the immune system in wild-type mice growing murine sarcoma cells (Cao et al., 2023). This broad effect and the fact that growing antlers have a variety of tissues (including skin, cartilage, bone, blood vessels and nerves) led (Landete-Castillejos et al., 2019; Wang and Landete-Castillejos, 2023) to suggest that deer antlers may have a general anticancer activity, particularly in the tips, the growing part. A comprehensive study by the same group (Rossetti et al., 2024), using the same DVA extract methodology, showed that DVA had direct anticancer effects in several human tumours (ten cell lines of glioblastoma, colon, breast and leukaemia). In addition to the direct effects in cell cultures, in mice with GBM, tumour weight was reduced by 61%–66% (oral-intraperitoneal administration, respectively) after 28 days of treatment, and the remaining tumour showed signs of liquefactive necrosis.

The variability in the methodology used to obtain the growing antler extract between studies calls for a study of the efficacy of the various antiseptic and disinfectant methods used to remove environmental microorganisms. However, it is important to avoid altering the proteins and other biomolecules that have anticancer properties (primary metabolites such as proteins, carbohydrates and lipids; or secondary metabolites such as terpenes, phenolics, glycosides and alkaloids). No study so far has evaluated different disinfection methods and their effect on the anticancer properties of the DVA obtained.

The removal of contaminants and pathogens from samples obtained for clinical use is crucial to ensure an effective response without causing negative side effects that may affect patients. Methods of pathogen removal can be physical: pressure, temperature, ultraviolet and gamma radiation, filtration; chemical: ethylene oxide, chlorinated compounds, ethane; enzymatic: lysozymes, proteases, oxidative enzymes (Bernal-Chávez et al., 2021; Paciulli et al., 2022). However, many of these options involve the degradation or modification of molecules and their properties, resulting in the loss of their functionality and biomedical properties. Examples of methods that significantly affect the antioxidant properties of such molecules are radiation and high temperatures (Carpena et al., 2021; Hosseini et al., 2022).

Therefore, the aim of our study was to determine, for the first time, an effective and efficient method of maintaining the asepsis of DVA without altering its active compounds. Therefore, in a first step, we evaluated nearly all of the most commonly used antiseptic and disinfection methods. In a second step, we evaluated the direct anticancer effects of the DVA extract obtained by each method on several cell culture lines belonging to the most common tumour type: colon cancer. In addition, since previous research (Rossetti et al., 2024) showed anticancer activity of orally administered DVA (a process that degrades the compounds), we propose a simulated digestion process to assess the direct anticancer effects in cell cultures of products derived from digested DVA.

Since sterility is essential for cell culture based experiments, the sterilization techniques evaluated in this study were selected to represent the most commonly applied physical and chemical approaches in laboratory practice, including filtration, heat exposure, ultraviolet radiation, and autoclaving. These methods differ in their potential impact on protein structure and molecular stability, which may consequently influence the measurable *in vitro* bioactivity of DVA extracts. Therefore, comparing these techniques allowed us to assess both their effectiveness in microbial control and their capacity to preserve extract integrity under standardized experimental conditions.

## 2 Materials and equipment

All materials used in this study is shown in Table 1. Other materials specific to this work can be found in Supplementary Material 1.

**TABLE 1:** Standard laboratory equipment for cell culture, protein analysis, and microscopy was used unless otherwise specified.

Equipment	Model	Supplier	Country
Blade mill	Retsch SM300	Retsch GmbH	Haan, Germany
Mixer mill	Retsch MM400	Retsch GmbH	Haan, Germany
Freeze-dryer	BIOBASE BK-FD10PT	BIOBASE BIODUSTY	Wolfenbüttel, Germany
Microplate reader	BIOBASE-EL 10th	BIOBASE BIODUSTY	Wolfenbüttel, Germany
Imaging system	ImageQuant LAS 500	GE Healthcare Life Sciences	Chicago, Illinois, USA
Cytometer	FACS Canto cytometer	Becton Dickinson	Franklin Lakes, Nueva Jersey, USA

Reagent	Supplier	Country
BCA Protein Assay Kit	MerckMillipore	Massachusetts, USA
Prestained Protein Ladder	abcam	Cambridge, UK
SureCast™ Gel Manual Preparation Pack A	Thermo Fisher Scientific	Waltham, Massachusetts, USA
Coomasie blue G-250 5 mg	Sigma Aldrich	Burlington, Massachusetts, USA
Thiazolyl Blue Tetrazolium Bromide (MTT)	BioChem, PanreacApplichem	Barcelona, Spain
Glyo-Fixx™	Thermo Fisher Scientific	Waltham, Massachusetts, USA

Antibody	Code	Supplier	Country
Anti-BCRP1	130-104-958	Miltenyi Company	Bergisch Gladbach, Germany
Anti-CD133	130-113-106	Miltenyi Company	Bergisch Gladbach, Germany
Anti-AC133	130-113-186	Miltenyi Company	Bergisch Gladbach, Germany
Anti-EPCAM	130-111-000	Miltenyi Company	Bergisch Gladbach, Germany
Anti-CD34	130-113-179	Miltenyi Company	Bergisch Gladbach, Germany
Anti-CD36	130-110-740	Miltenyi Company	Bergisch Gladbach, Germany
Anti-CD44V6	130-111-238	Miltenyi Company	Bergisch Gladbach, Germany
Anti-TROP2	130-115-055	Miltenyi Company	Bergisch Gladbach, Germany
Anti-CD44	130-114-535	Miltenyi Company	Bergisch Gladbach, Germany
Anti-DCLK1	ab202755	abcam	Cambridge, UK
Anti-RAGE	Ab237363	abcam	Cambridge, UK
Anti-LGR5	562912	Becton Dickinson (BD)	Franklin Lakes, Nueva Jersey, USA
Anti-CD166	559263	Becton Dickinson (BD)	Franklin Lakes, Nueva Jersey, USA

Program	Version	Supplier	Country
ImageJ	1.46j	National Institutes of Health (NIH)	Bethesda, Maryland, USA
GraphPad Prism	8.0.1	GraphPad Software Inc.	San Diego, CA, USA
FlowJo	10.8.1	Becton Dickinson (BD)	Franklin Lakes, Nueva Jersey, USA

## 3 Methods

### 3.1 Material acquisition and antler sections

Four growing antlers from adult red deer (*Cervus elaphus*) were obtained from UCLM deer experimental farm under anaesthesia and with protocols approved by the ethical committee of the UCLM (CEEA: PR-2022-09). The stages of antler growth of the males were chosen to be similar to growth antlers in mid cycle, 60 days after casting, based on our experience in the experimental deer of the UCLM and guidelines from Deer Industry New Zealand.

Prior to antler removal, animals were sedated by intramuscular administration of a mixture of xylazine (Xilagesic®, Laboratory Calier, Spain; 0.4 mg/kg body weight) and ketamine (Imalgene®, Merial, France; 1 mg/kg body weight) to ensure adequate immobilization (hydraulic immobilizer in the handling bay) before the cutting procedure. Local subcutaneous anesthesia was achieved by infiltration of lidocaine (20 mg/mL; approximately 20 cm per animal at the cutting site). Additionally, analgesia was provided with subcutaneous carprofen (1.5 mg/kg body weight) at the time of antler sectioning. The antlers were initially cleaned with soap and water, and the cutting area was subsequently disinfected with povidone-iodine. Once the area was prepared, rubber tourniquets were placed around the base of the antlers to prevent bleeding. The antlers were cut 2 cm above the base using an electric saw (Kebonds mechanical saw, Parkside, Saxony, Germany). Hemostasis was immediately achieved after antler sectioning by thermal cauterization of the cutting site to prevent bleeding and ensure animal safety. Hemostasis was immediately achieved after antler sectioning by thermal cauterization of the cutting site to prevent bleeding and ensure animal safety. To prevent infection and promote healing, chlortetracycline and healing powder (Neocur) were administered, and then the tourniquets were removed. All procedures were performed under veterinary supervision to ensure animal welfare and minimize pain or distress.

Once the area was prepared, the antlers were cut at the base using an electric saw (Kebonds mechanical saw, Parkside, Saxony, Germany). The collected samples were stored at −80 °C until sections were obtained from the tip, middle, and base of the antler. These sections were then lyophilised at 0.2 mbar for 46 h (condenser temperature, −45 °C) (Sublimator 30 EKS, ZIRBUS technology GmbH, Bad Grund, Germany). After lyophilisation, they were ground with a blade mill at 1500 rpm and 2 mm pore size (Retsch SM300, Retsch GmbH, Haan, Germany) and then mixed with a mixer mill at 30 Hz for 2 min until a density of 0.18 mm was reached (Retsch MM400, Retsch GmbH, Haan, Germany).

For this study, 4 bases and 4 antler tips from 4 different stags were used from the main beam, as shown in Figure 2B. Antler sections were standardized across animals by cutting approximately 5 cm from the distal growing tip and 15 cm from the basal region of the main beam, ensuring comparable anatomical regions among specimens.

### 3.2 Compounds extraction

Lyophilised powder from growing adult deer antlers was mixed with sterile Milli-Q water (Ultramatic GR, Wasserlab, Navarra, Spain) in a ratio of 1:10 (p/v) by magnetic stirring for 1 h at room temperature (SBS-MR-1600/6, Expondo, Berlin, Germany; Alegría-Aravena et al., 2025). The samples were then centrifuged at 8500 *g* for 20 min at 4 °C (UNIVERSAL 320, Hettich, Tuttlingen, Germany). The supernatant was separated into two parts, one of which was frozen in liquid nitrogen and lyophilised for 48 h (BIOBASE BK-FD10PT, BIOBASE BIODUSTY, Shandong, China). Both extracts from 1 g of deer velvet antler were normalized to 1X PBS solution.

### 3.3 Sterilisation extract

Aliquots of each extract (lyophilised and non-lyophilised) were subjected to different sterilisation methods: filtration through polyethersulfone (PES), cellulose acetate (CA), mixed cellulose ester (MCE) and Regenerated cellulose (RC) membranes of 0.22 µm; fast pasteurisation by exposure to 72 °C for 15 s (FP); slow pasteurisation by exposure to 60 °C for 30 min (SP); exposure to UV radiation for 15 min at a distance of 54 cm from the radiation source (UV); autoclaving (A); no treatment (NT).

### 3.4 Extract digestion

To monitor the degradation of the extract, an aliquot of lyophilised and non-lyophilised extract was subjected to digestion. The gastric buffer contains 0.52 g of Potassium chloride; Monopotassium phosphate of 0.12 g; 2.1 g of Sodium hydrogen carbonate; 2.76 g of Sodium chloride; 0.02 g of Magnesium chloride hexahydrate; 0.08 g of Ammonium carbonate; 0.02 g of Calcium chloride dihydrate; 0.04 g of Pepsin per liter. Gastric simulation buffer containing pepsin proteinase adjusted to pH 2.5 was added at a ratio of 1:1 (v/v), shaken for 2 h and the digested extract stored at −80 °C.

### 3.5 Aseptic control of samples

#### 3.5.1 Growth of microorganisms in DMEM culture medium

The level of growth of microorganisms was determined by inoculation of the extract into 96-well microtiter plates with culture medium for animal eukaryotic cells at 0.5 mg/mL of protein samples. For this purpose, DMEM medium (Dominique Dutscher, Bernolsheim, France) without antibiotic, supplemented with 10% Fetal Bovine Serum (FBS, Pan BioTech, Aidenbach, Germany) and 1% glutamine (Pan BioTech, Aidenbach, Germany) was used. The samples were incubated for 72 h at 37 °C. Finally, the wells were photographed at ×20 magnification (Motic AE2000, MoticEurope, Barcelona, Spain), and absorbance was measured at 562 nm in a plate reader (BIOBASE-EL 10th BIOBASE BIODUSTY, Wolfenbüttel, Germany). As a negative control, DMEM medium without antibiotic supplemented with 10% Fetal Bovine Serum and 1% glutamine was used as a negative control.

#### 3.5.2 Growth of microorganisms in LB culture medium

In test tubes with liquid LB medium (LB Lennox, Labbox Labware S.L., Barcelona, Spain), sterilised samples were inoculated with the different methods at 0.5 mg/mL of protein samples and then shaken at 150 rpm for 24 h at room temperature. Finally, the samples were transferred to a microtiter plate and the absorbance was measured at 630 nm to check bacterial growth on a plate reader, LB medium without inoculum was used as a negative control.

### 3.6 Protein Assessment

#### 3.6.1 Protein quantification

Protein concentration was determined by BCA Protein Assay Kit (MerckMillipore, Massachusetts, USA). A BSA standard line was used with the points of 2, 1, 0.5, 0.25, 0.125, 0.0625, 0.03125, 0.015625 and 0 mg/mL. Samples were diluted at a ratio of 1:50 (v/v) to fall within the interpolable range of the reference curve.

The proteinase enzyme contained in the digestion buffer reacts with the kit used to generate a signal (colour). For this reason, a control was performed by adding the same amount of digestion buffer and then normalized by subtracting its signal from the digested sample within the assay. The reaction was measured at 562 nm using a microplate reader.

#### 3.6.2 Gel visualisation

To observe possible degradability of the samples, they were visualized by polyacrylamide gel at 4%–12%. 10µg of total proteins were loaded after denaturation at 96 °C. Prestained Protein Ladder (ab116028, abcam, Cambridge, UK) was used as molecular weight marker. Electrophoresis was carried out in 1X MES-SDS buffer (Life Technologies, California, USA) at 100 V for 1.5 h. Subsequently, Coomassie staining was used for 2 h and washed overnight in destaining solution at 4 °C under constant agitation.

The relative percentage showing the presence of each protein band was measured on the gel images using NIH ImageJ software (Rueden et al., 2017). High resolution images of polyacrylamide gel at 4%–12% were analyzed by selecting each protein band with the rectangle tool, ensuring background was included for proper subtraction. The pixel intensity of each band was quantified using the “Plot Profile” function to determine the peak and calculate the area under the curve corresponding to the band’s intensity. Background intensity was measured in an empty region of the gel and subtracted from each band’s intensity to ensure accuracy. The relative percentage of each protein band was calculated by dividing its intensity by the total intensity of all bands in the same lane, multiplying by 100.

### 3.7 Evaluation of DVA extracts anticancer activity

#### 3.7.1 Viability of tumour cells

**FIGURE 1:**
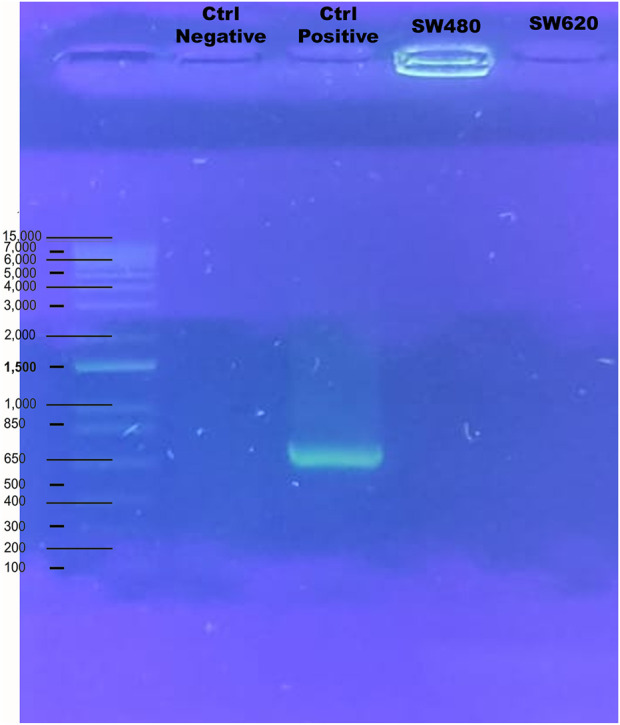
PCR of *mycoplasma* detection in cell lines. These lines were cultured and expanded in DMEM-High Glucose, with 10% FBS, 2 mM L-glutamine, and 1% penicillin/streptomycin at 37°C in humidified incubator with 5% CO2 (Series II water Jacker, Thermo Scientific, Waltham, MA, USA).

The effects were studied in four cell lines from two different types of cancers. Colorectal cancer cell line SW480 and SW620 obtained from the American Type Culture Collection (ATCC, Manassas, VA, USA). Table 2 shown the different characteristics and specification of cell lines used in this work, and Figure 1 shown the verification of the cell lines free *mycoplasma* by PCR technique.

**TABLE 2:** Characteristics of cell lines.

*Official cell line name*	Species	Tissue of origin	Sex	Research resource identifier (RRID)	Source/Supplier	Date of obtain	Mycoplasma contamination
SW480	*Homo sapiens* (human)	Colorectal adenocarcinoma (primary tumor, colon)	Male	RRID:CVCL_0546	ATCC (CCL-228)	02/2023	No contamination
SW620	*Homo sapiens* (human)	Lymph node metastasis derived from colorectal adenocarcinoma (same patient as SW480)	Male	RRID:CVCL_0547	ATCC (CCL-227)	02/2023	No contamination

A microtiter plate dose-response assay was performed to establish the level of cell cytotoxicity to the different sterilised samples. 100,000 cells/mL were seeded and incubated for 16 h at 37 °C with 5% CO2. Subsequently, 1 mg/mL of the different extracts were added and DMEM culture medium was used as a negative control. This concentration was selected from our previous studies where up to 40% cell viability was achieved (Chonco et al., 2021; Rossetti et al., 2024). The cells were exposed to treatment for 72 h at 37 °C with 5% CO2. After 72 h of exposure to treatment, the cells were revealed by thiazolium blue tetrazolium bromide at 500 μg/mL to detect cell viability MTT assay (BioChem, PanreacApplichem, Barcelona, Spain). The reagent was incubated with the cells for 4 h at 37 °C with 5% CO2 until formazan crystals formed. Finally, the crystals were solubilized with DMSO (Labbox Labware, SL, Barcelona, Spain). The reading was performed at 542 nm in a spectrophotometer.

#### 3.7.2 Tumour biomarkers by flow cytometry analysis

The different biomarkers expressed by the cell lines were analysed by flow cytometry. Anti-BCRP1, anti-CD133 (1/5), anti-AC133 (1/5), anti-EPCAM (1/5), anti-CD34, anti-CD36 (1/5), anti-CD44V6 (1/5), anti-TROP2 (1/10), and anti-CD44 (1/5); antibodies were purchased from Miltenyi Company (Bergisch Gladbach, Germany), anti-DCLK1 (1/5), and anti-RAGE (1/5) antibodies were purchased from abcam (Cambridge, UK); anti-LGR5 and anti-CD166 antibodies were purchased from Becton Dickinson (Franklin Lakes, NJ, USA).

A quantity of 105 cells/well was seeded in 12 well plates and incubated with the extracts from deer antler tip and base, lyophilised and non-lyophilised, filtered with PES membrane for 72 h. Then, the cells were disaggregated with trypsin and resuspended in PBS + BSA 0.5% (Sigma-Aldrich, St. Louis, MO, USA). Incubation with antibody was carried out for 15 min at room temperature in the dark. Cells were then washed and fixed with Glyo-FixxTM (Thermo Fisher, Massachusetts, USA). Finally, cell labelling was detected by FACS Canto cytometer and the data analyse was realized with FloJo v10.8.1 (both from Becton Dickinson, Franklin Lakes, Nueva Jersey, USA). Flow cytometry analysis included sequential gating to exclude debris and doublets based on FSC/SSC and FSC-A/FSC-H parameters (Supplementary Material 2).

### 3.8 Statistical analysis

GraphPad Prism 8.0.1 (GraphPad Software Inc., San Diego, CA, USA) was used for statistical analysis with one-way parametric analysis of variance (ANOVA) to compare normally distributed groups and non-parametric analysis for outliers. Tukey’s *post hoc* test was employed for multiple comparisons, the statistical level was considered 0.05. All graphs shown have been made with 8 biological replicates in quadruplicate. Data are presented as mean ± standard error of the mean (SEM). The significant differences are indicated as ***p < 0.001, **p < 0.01, *p < 0.05.

## 4 Results

### 4.1 Antlers sections and lyophilisation of deer antler

**FIGURE 2:**
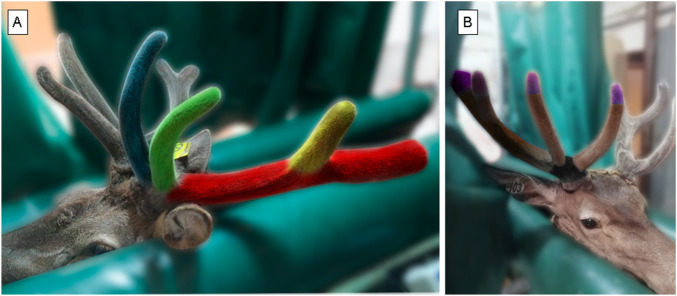
Segments of deer antler. **(A)** Antler bifurcations. Red, yellow, green and blue represent the main beam and Trez, Bez and Brow bifurcations, respectively. **(B)** Antler sections: TIP, MID and BASE. Example of a deer antler used in the study, divided into three zones: TIP (violet, 2.5 cm), MID (brown, middle zones of 5 cm), and BASE (rest of the antler, black). The sample was obtained at 60 days of growth, at which point it is harvested for experimental use.

Four growing antlers of adult red deer (*Cervus elaphus*) of 60 days after casting were weight before and after the lyophilisation process as described in Materials and Methods. The antlers are implanted on a bony pivot by a wide and very rough base, which is the rosette. From a central stem, the different bifurcations, called tines, are formed. The first, the Brow tine, comes out vertically and slightly outwards (blue). Above the Brow is the Bez (green). In the middle of the stem is the Trez (yellow). At the upper end, at 60 days of growth as shown in Figure 2A, the central part is called the main beam (red). Later on, depending on the number of tips, there is a fork (with two tips) or a palm (more than two tips). A palm rich in tips forms the crown. Figure 2B details the antler sections estimated for each bifurcation.

The weights of the whole antler and the sections used in the paper are given in Table 3 below. After freeze-drying, an average water loss of 71.5% was achieved (Total antler). Noteworthy, the greatest loss occurred at the tip of the antler (82%, where it grows and has the highest concentration of soft tissue) compared to the base (66.7%, more mineralised).

**TABLE 3:** Weight of antlers before and after lyophilisation.

Antler section	Weight before lyophilisation (g)	Weight after lyophilisation (g)
Beam TIP	50 ± 5	9.1 ± 0.9
Beam MID1	132 ± 10	29 ± 1
Base	336 ± 32	112 ± 10
Total	1196 ± 57	341 ± 13

### 4.2 Growth of microorganisms in sample

**FIGURE 3:**
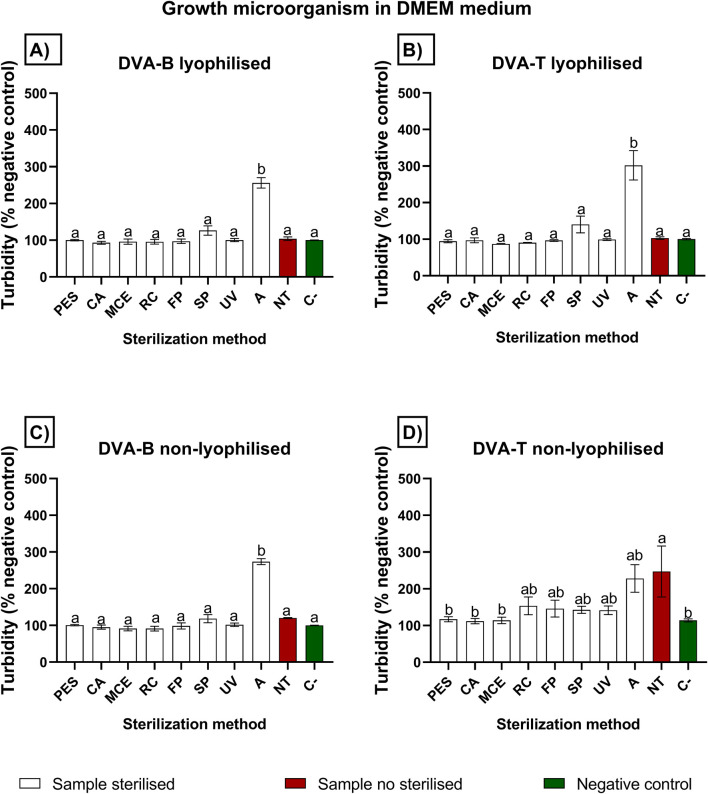
Growth microorganism in DMEM medium. Values are expressed as percentage relative to the negative control (C−), which was set at 100%. Increased values reflect turbidity and do not necessarily indicate microbial proliferation, particularly in autoclaved samples. Evaluation of different sterilisation methods for protein extracts from deer antler filtration through polyethersulfone (PES), cellulose acetate (CA), mixed cellulose ester (MCE) and Regenerated cellulose (RC) membranes of 0.22 µm; fast pasteurisation by exposure to 72°C for 15 s (FP); slow pasteurisation by exposure to 60°C for 30 min (SP); exposure to UV radiation for 15 min at a distance of 54 cm from the radiation source (UV); autoclaving **(A)**; no treatment (NT). The graphs represent turbidity mesure in absorbance at 524 nm of DMEM medium incubated for 72 h with **(A)** DVA BASE: lyophilised antler base extract post protein extraction and before sterilisation; **(B)** DVA TIP: lyophilised antler tip extract post protein extraction and before sterilisation; **(C)** non-lyophilised antler base extract; **(D)** non-lyophilised antler tip extract. The reference value is the negative control (C-) in green columns, which is DMEM medium alone without any possibility of microorganism contamination (green bar). The no treatment (NT) in red-brown columns is the extract without sterilisation method. A higher absorbance indicates turbidity produced by different levels of microorganism growth. Acronyms stand in 2.3 section. Data are presented as mean ± SEM (n = 8). The significant differences are indicated as bars data not sharing the same letter are significantly different (p < 0.05) between sterilisation methods.

**FIGURE 4:**
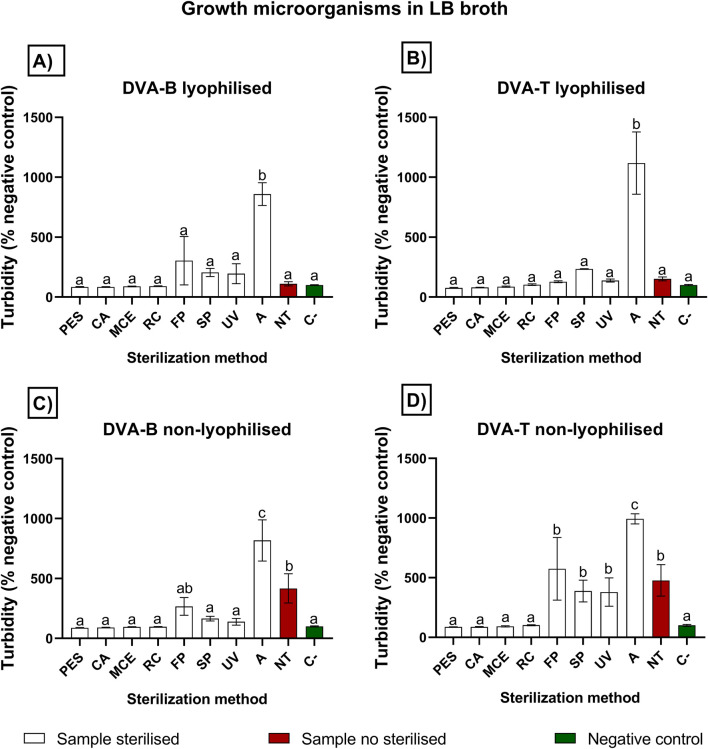
Growth microorganisms in LB broth. Values are expressed as percentage relative to the negative control (C−), which was set at 100%. Evaluation of different sterilisation methods for protein extracts from deer antler filtration through polyethersulfone (PES), cellulose acetate (CA), mixed cellulose ester (MCE) and Regenerated cellulose (RC) membranes of 0.22 µm; fast pasteurisation by exposure to 72°C for 15 s (FP); slow pasteurisation by exposure to 60°C for 30 min (SP); exposure to UV radiation for 15 min at a distance of 54 cm from the radiation source (UV); autoclaving **(A)**; no treatment (NT). The graphs represent absorbance at 630 nm of LB broth culture incubated for 24 h with **(A)** lyophilised antler base extract post protein extraction and before sterilisation; **(B)** lyophilised antler tip extract post protein extraction and before sterilisation; **(C)** non-lyophilised antler base extract; **(D)** non-lyophilised antler tip extract. The reference value is the negative control (C-) in red color, which is LB culture medium alone without any possibility of microorganism contamination (green bar). The no treatment (NT) in red-brown columns is the extract without sterilisation method. A higher absorbance indicates turbidity produced by different levels of microorganism growth. Data are presented as mean ± SEM (n = 8). The significant differences are indicated as bars data not sharing the same letter are significantly different (p < 0.05) between sterilisation methods.

**FIGURE 5:**
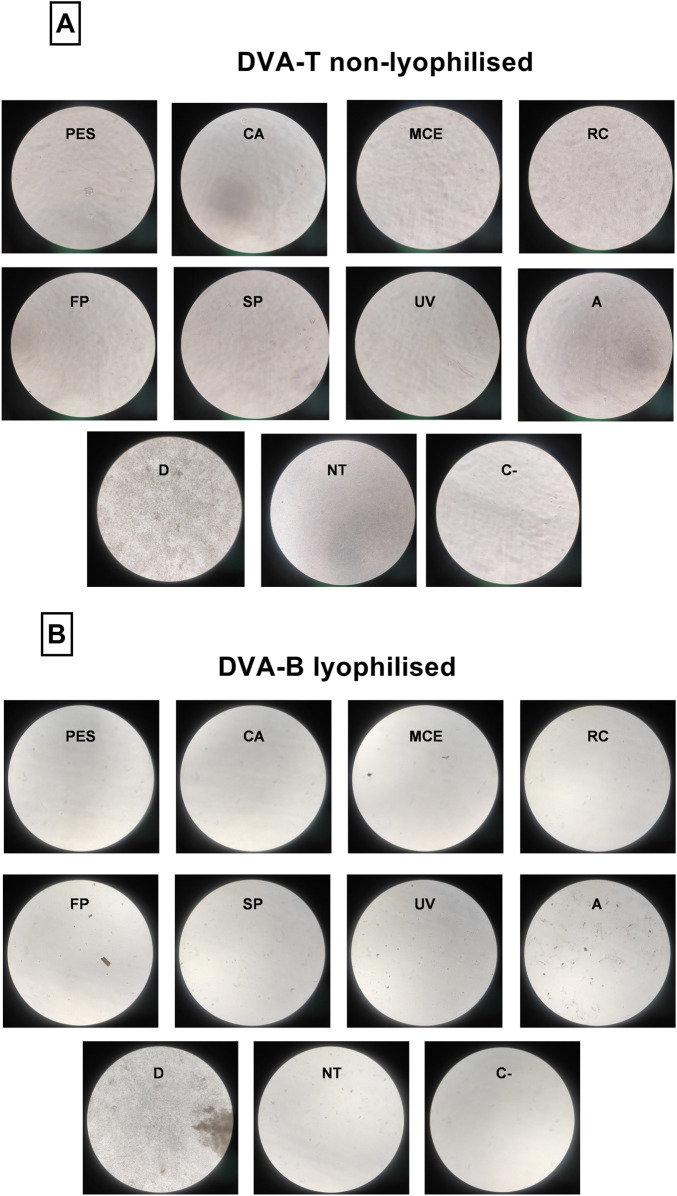
Micrographs of microorganism growth in DMEM medium. **(A)** corresponds to non-lyophilised TIP samples, while **(B)** are lyophilised Base samples. The sterilization methods are filtration through polyethersulfone (PES), cellulose acetate (CA), mixed cellulose ester (MCE) and Regenerated cellulose (RC) membranes of 0.22 µm; fast pasteurisation by exposure to 72°C for 15 s (FP); slow pasteurisation by exposure to 60°C for 30 min (SP); exposure to UV radiation for 15 min at a distance of 54 cm from the radiation source (UV); autoclaving **(A)**; digested sample (D); no treatment (NT); only culture medium (C-).

The quantification of bacterial growth by spectrophotometry is illustrated in Figures 3, 4, which depict the results obtained with Dulbecco’s Modified Eagle Medium (DMEM) and Luria-Bertani broth (LB), respectively. Regarding the DMEM culture medium, all the methods yielded a comparable absorbance value to that of the negative control (sterile culture medium assessed directly from the bottle), thereby demonstrating the efficacy of these methods in preventing microbial growth. It is noteworthy that lyophilised and non-lyophilised samples subjected to autoclaving resulted in a turbid solution which, upon contact with the culture medium, prevented absorbance analysis for the presence of microorganisms. In contrast, non-treated samples led to microorganism growth when they were not lyophilised. However, lyophilisation appeared to kill microorganisms, as untreated samples did not result in microorganism growth if they were lyophilised. The micrographs were shown in Figure 5.

### 4.3 Protein quantification

**FIGURE 6:**
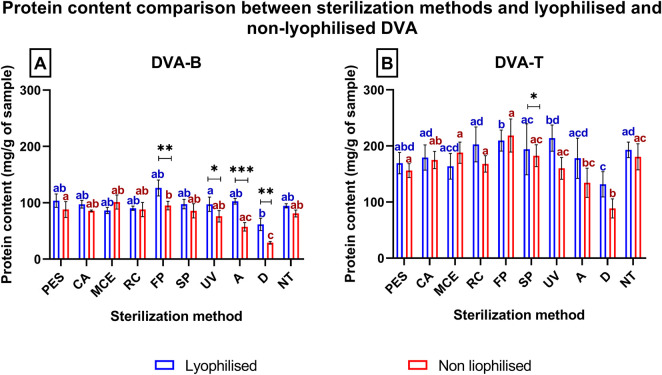
Comparison of protein content of lyophilised (bars) and non-lyophilised deer antler extract (red dots), after different sterilisation methods, and in case of simulation of digestion. The graphs represent the content in milligram (mg) of protein obtained per Gram of sample from **(A)** DVA-B; **(B)** DVA-T. The sterilization methods are filtration through polyethersulfone (PES), cellulose acetate (CA), mixed cellulose ester (MCE) and Regenerated cellulose (RC) membranes of 0.22 µm; fast pasteurisation by exposure to 72°C for 15 s (FP); slow pasteurisation by exposure to 60°C for 30 min (SP); exposure to UV radiation for 15 min at a distance of 54 cm from the radiation source (UV); autoclaving **(A)**; no treatment (NT). Data are presented as mean ± SEM (n = 8). The significant differences are indicated as ***p < 0.001, **p < 0.01, *p < 0.05 for differences between lyophilisation; and bars data not sharing the same letter are significantly different (p < 0.05) between sterilisation methods, liophilised in blue and no liophilised in red.

To ascertain whether a loss of protein occurs during the sterilisation of the extract, the total protein content was quantified. Figure 6 presents a comparison of the protein content per Gram of sample obtained after sterilisation using the various methods under consideration. A comparison of the lyophilised and non-lyophilised procedures revealed that the DVA from the Base (DVA-B) exhibited a higher protein content after lyophilisation and sterilisation by FP, UV, A, and D (Figure 6A), with a difference of 25% ± 6%, 22% ± 10%, 44% ± 7%, and 53% ± 4%, respectively. Similarly, DVA from the Tip (DVA-T) after lyophilisation and UV sterilisation demonstrated a difference of 23% ± 9% in comparison to the non-lyophilised sample (Figure 6B).

In contrast, when the sterilised lyophilised samples were compared with the untreated samples, it was observed that none of them lost protein, except for the digested sample. In the case of non-lyophilised samples, the digested and autoclaved samples exhibit notable differences in comparison to the non-sterilised control.

### 4.4 Protein visualization

**FIGURE 7:**
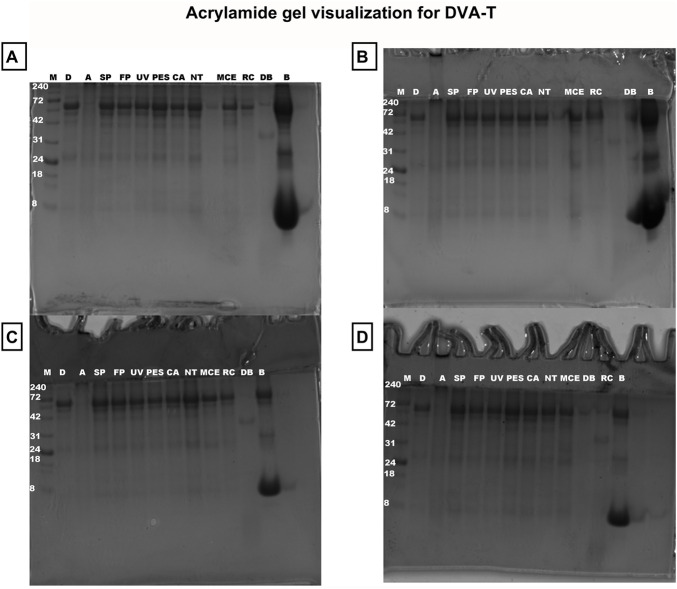
Acrylamide gel visualization for DVA-T. Samples non lyophilised where **(A–D)** represent independent assays. The carriers is marker (M); ample with digestion **(D)**; autoclaving **(A)**; slow pasteurisation by exposure to 60°C for 30 min (SP); fast pasteurisation by exposure to 72°C for 15 s (FP); exposure to UV radiation for 15 min at a distance of 54 cm from the radiation source (UV); filtration through polyethersulfone (PES), cellulose acetate (CA), mixed cellulose ester (MCE) and Regenerated cellulose (RC) membranes of 0.22 µm; no treatment (NT); digestion buffer (DB); charge control, blood **(B)**.

**FIGURE 8:**
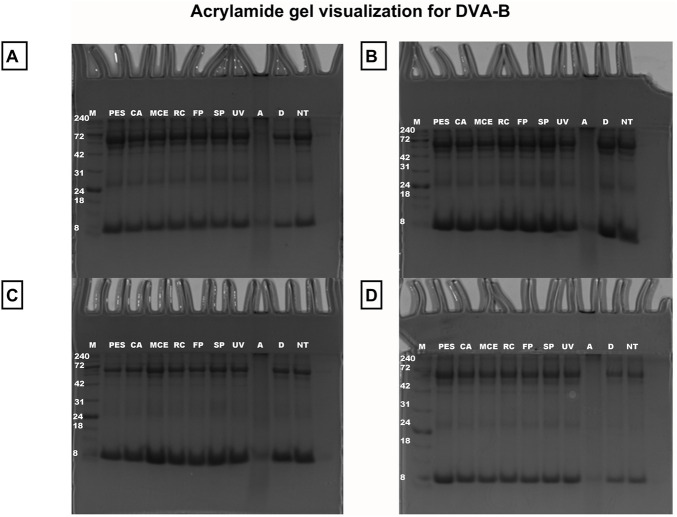
Acrylamide gel visualization for DVA-B. Samples lyophilised where **(A–D)** represent independent assays. The carriers is marker (M); ample with digestion **(D)**; autoclaving **(A)**; slow pasteurisation by exposure to 60°C for 30 min (SP); fast pasteurisation by exposure to 72°C for 15 s (FP); exposure to UV radiation for 15 min at a distance of 54 cm from the radiation source (UV); filtration through polyethersulfone (PES), cellulose acetate (CA), mixed cellulose ester (MCE) and Regenerated cellulose (RC) membranes of 0.22 µm; no treatment (NT); digestion buffer (DB); charge control, blood **(B)**.

**FIGURE 9:**
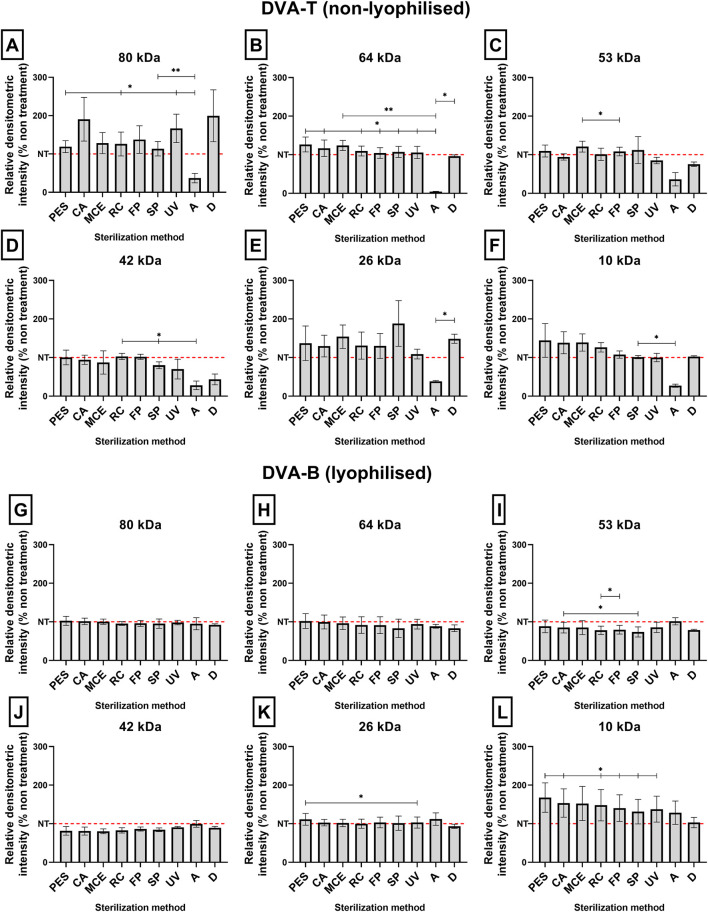
Twelve bar charts show relative densitometric intensity (% of non-treatment control) of protein bands after electrophoresis. Panels **(A–F)** correspond to non-lyophilised DVA-T samples, while panels **(G–L)** correspond to lyophilised DVA-B samples. Each panel represents proteins of different molecular weights (80, 64, 53, 42, 26, and 10 kDa). Bars compare multiple sterilisation methods, with the control level (no treatment) indicated by a dashed red line. Data are presented as mean ± SEM (n = 8). Asterisks indicate statistically significant differences (p < 0.05, **p < 0.01). Variations in intensity reflect changes in protein content depending on treatment and sample type.

**FIGURE 10:**
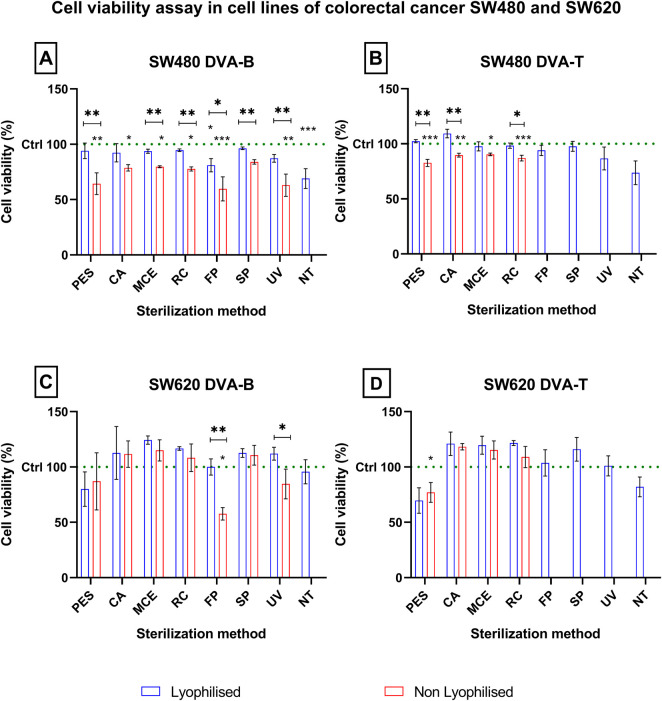
Cell viability assay in cell lines of colorectal cancer SW480 and SW620 after 72 h exposure to DVA (assessed by the MTT method). The viability of negative control (no anticancer extract applied) is the green dots line (C-, negative control). The sterilization methods are filtration through polyethersulfone (PES), cellulose acetate (CA), mixed cellulose ester (MCE) and Regenerated cellulose (RC) membranes of 0.22 µm; fast pasteurisation by exposure to 72°C for 15 s (FP); slow pasteurisation by exposure to 60°C for 30 min (SP); exposure to UV radiation for 15 min at a distance of 54 cm from the radiation source (UV); no treatment (NT). The letter labelling each graphic on their upper left stands for, respectively: **(A)** SW480 cell line exposed to lyophilised and non-lyophilised DVA BASE, **(B)** SW480 cell line exposed to lyophilised and non-lyophilised DVA TIP, **(C)** SW620 cell line exposed to lyophilised and non-lyophilised DVA BASE, **(D)** SW620 cell line exposed to lyophilised and non-lyophilised DVA TIP. Significant differences are shown in two comparisons: those above each bar indicate level of significance in viability after treating with either lyophilised (blue) or non-lyophilised (red) for that particular treatment. Data are presented as mean ± SEM (n = 8). Level of significance indicated as *** for p < 0.001, ** for p < 0.01 and * for p < 0.05.

Following the quantification of the protein content of each sample, the samples were visualized on a polyacrylamide gel. The various sterilisation techniques demonstrated no evidence of degradation (Figures 7, 8). However, the autoclaved extract failed to pass through the gel, thereby confirming the degradation process observed earlier when analysing the growth of microorganisms in DMEM and LB medium (see Figures 4, 5). For this reason, autoclaved extract is not shown in Figure 10. In comparison to the untreated sample, the digested samples exhibited a loss of bands. The protein fraction with a molecular weight of 42 kDa decreased by 64% ± 12%, while the protein fraction with a molecular weight of 53 kDa decreased by 25% ± 5% (see Figure 9).

### 4.5 Cell viability

The impact of the contamination-free extracts was evaluated on colorectal cancer cell lines (Figure 10) through cell viability assays. The findings indicate a more pronounced reduction in cell viability in non-lyophilised extracts relative to lyophilised extracts across all sterilisation treatments (autoclaving excluded) when compared to the negative control (100% viability, represented by the green bar).

The results for the SW480 line (Figures 10A,B) demonstrate a more pronounced effect than those observed in the SW620 line. Therefore, the antler lyophilised sample without additional treatment of the extract (untreated, NT) was observed to elicit the most pronounced antitumour effect, both in DVA-T (with a decrease of up to 37% ± 10%) and in DVA-B (with a decrease of up to 69% ± 8%). In the case of the treatment that did not involve the lyophilisation of the extract (non-lyophilised, black dots), most samples demonstrated an antitumour effect, with DVA-B resulting in cell viability of up to 59% ± 10%. However, regarding DVA-T, it was observed that only the sample filtered with a PES membrane exhibited an effect, with a decrease in cell viability of up to 82% ± 3%. In the SW620 cell line (see Figures 10C,D), the DVA-B samples that underwent lyophilisation exhibited no discernible effect. In contrast, the non-lyophilised samples that underwent rapid pasteurisation demonstrated a notable reduction in cell viability, reaching a maximum of 57% ± 5%. Conversely, samples from DVA-T only demonstrated activity on the lyophilised and non-lyophilised DVA extract when subjected to PES filtration, resulting in a viability of 77% ± 8%.

### 4.6 Tumour biomarkers

**FIGURE 11:**
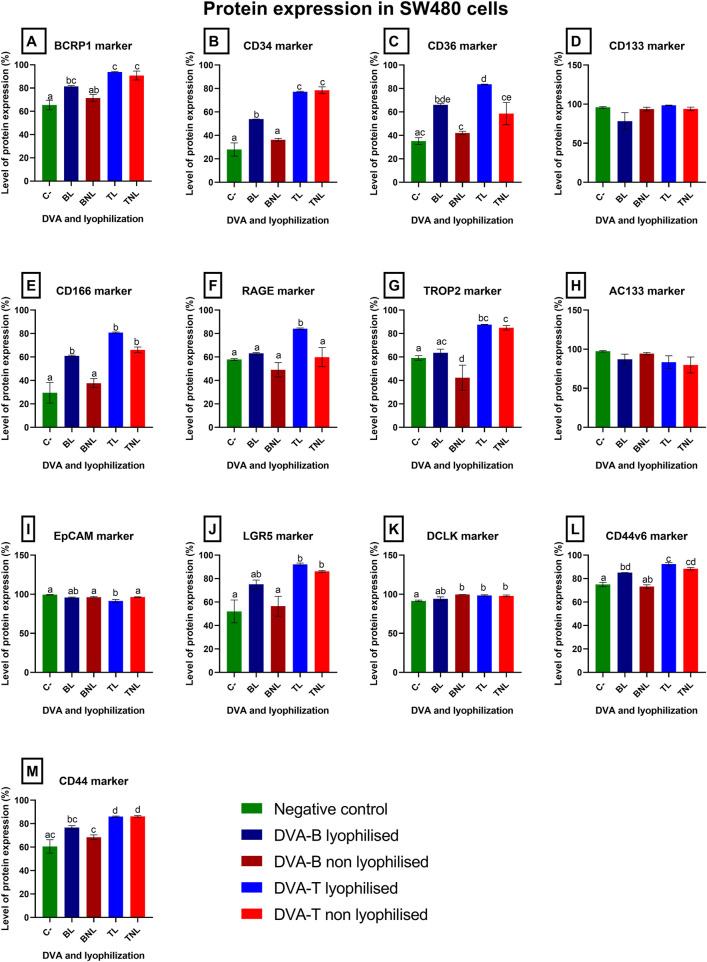
A grid of bar charts Panels **(A–M)** shows protein expression levels in SW480 cells after 72 hours of exposure to deer antler extracts (DVA). Each panel represents a different biomarker (BCRP1, CD34, CD36, CD133, CD166, RAGE, TROP2, AC133, EpCAM, LGR5, DCLK1, CD44v6, and CD44). Bars compare five groups: negative control (green), DVA-B lyophilised (blue), DVA-B non-lyophilised (dark blue), DVA-T lyophilised (red), and DVA-T non-lyophilised (dark red). The y-axis indicates the percentage of cells expressing each protein. Data are presented as mean ± SEM (n = 8). Different letters indicate statistically significant differences (p < 0.05).

Given that the principal anticancer effect of DVA was identified in the SW480 colorectal tumour cell line, we conducted a molecular analysis by flow cytometry of the primary biomarkers associated with tumour resistance and progression, in addition to assessing biomarkers linked to stem cell and tissue regeneration. Figure 11 illustrates the distinction in the principal membrane proteins of the colorectal line subjected to extracts lyophilised and non-lyophilised from antler sections DVA-B and DVA-T filtrated by PES membrane. In general, both DVA extracts produced an increase in the percentage of cells expressing the markers with respect to the control, with a greater effect observed with the tip (the region in which the antler grows, DVA-T) than with the base (DVA-B).

The greatest effects of DVA-T (non-lyophilised) were observed in the increase effect in biomarkers: BCRP1 (90% ± 4%), CD34 (78% ± 3%), CD166 (66% ± 2%), Trop2 (85% ± 2%), LGR5 (86% ± 1%), CD44 (86% ± 1%). DVA-T (lyophilised) exhibited also an increase in BCRP1 (93.8% ± 0.4%), CD34 (77.2% ± 0.4%), CD36 (83.5% ± 0.3%), CD166 (80.8% ± 0.5%), RAGE (84% ± 1%), Trop2 (87.6% ± 0.2%), LGR5 (92% ± 1%), CD44 (86% ± 1%).

DVA-B (lyophilised) increase the expression of BCRP1 (81% ± 1%), CD34 (53.9% ± 0.1%), CD36 (66% ± 1%), CD166 (61% ± 1%), CD44 (77% ± 2%) and DVA-B (non-lyophilised) decreased the expression of TROP2 (71% ± 10%).

## 5 Discussion

It is of paramount importance to maintain the sterility of samples undergoing *in vitro* analysis, particularly when working with human cell lines, in order to prevent contaminationaffecting the efficiency of the bioactive molecules tested (Tao et al., 2021). However, as the sterilisation method may alter the proteins produced, potentially affecting the anticancer effect, it is also essential to evaluate how these methods interact with protein effects to identify the most effective and least damaging method for preserving protein integrity. This is particularly relevant considering that the number of peptides identified in deer antler with anti-tumour activity published (Hu et al., 2015; Yang et al., 2017; Cao et al., 2023; Sun et al., 2024), reached already 405 peptides with multiple biomedical applications (Li et al., 2022). A number of proteins present in growing deer antler tissues have been identified as being involved in cell proliferation and differentiation, and as potentially regulating anticancer properties. These include PEDF and CDKN1C (Lord et al., 2007). Furthermore, other protooncogenes (FOS, FAM83A, and REL) and tumour suppressor genes (PML, TP53 pathway related, and ADAMTS family members) are positively selected in cervids and highly expressed in the antler (Zhang et al., 2022). Specifically, p53 is one of the most amplified genes in various animal species, including elephants, where tumorigenic events are rarely documented (Tollis et al., 2017). The potential involvement of other tumour suppressors, such as FBXW7, is particularly relevant, given their overlapping functions, which may include a role upstream from p53 in protecting the genome and preventing tumour events (Shen et al., 2022). Therefore, it is crucial to maintain sterility in order to ensure the applicability of the method across different industries, including food and pharmacology (Akers et al., 1988; Gouveia et al., 2015). The analysis of environmental samples, whether derived from a plant or animal source, is contingent upon the consideration of diverse microbial populations that are capable of proliferation when exposed to conditions allowing their growth and development (Samarajeewa, 2023). In this regard, the continued presence of environmental pathogens may be influenced by the consumption or utilisation of the material, potentially leading to the emergence of opportunistic pathogen diseases (Koirala et al., 2023).

The results demonstrate that the autoclaving process may affect the physicochemical properties and biological activity of DVA hindering its application for the investigation of *in vitro* effects employing the methods described in this manuscript. A more thorough analysis employing techniques such as LC-MS/MS would be necessary to evaluate how high pressure and temperature affect the DVA extract (Leitzen et al., 2021). The freeze-drying process after antler removal at our experimental animal farm at 60 days of growth allowed us to further process the sections at any time of the year. Is it noteworthy that the remaining treatments did not demonstrably improve sterilisation beyond this initial stage. Specifically, no treatment yielded results that differed from the negative control. Additionally, the protein content of the samples was, in the majority of cases, comparable to that of the untreated extract. The reduction in viability produced by the different methods of sterilisation of DVA also demonstrated that the non-treated sample retained the most effective anticancer properties of the antler extract. Indeed, the majority of methods proved ineffective in further reducing the viability of tumour cells after treatment with DVA. DVA increased the number of tumour cells expressing the marker proteins in their membrane, with the greatest increase observed after applying samples from the TIP compared to those from the BASE. These results indicate that the optimal strategy for sterilising DVA samples without compromising their anticancer effect may be through the use of alternative methods.

Deer antler is increasingly being studied for its health properties (Sun et al., 2024). Around 20 publications in DOAJ (Directory of Open Access Journals), 150 in Pubmed as well as Scopus and NLM (National Library of Medicine), 540 in PMC (PubMed Central) use deer antler extract to investigate its wide range of properties, including bone regeneration (Kim et al., 2005; Ren et al., 2019), immunostimulatory (Kim et al., 2004), and anticancer properties (e.g., Tang et al., 2019; Zheng et al., 2020; Chonco et al., 2021; Rossetti et al., 2024). This is a challenge because antler extracts are obtained from a complex tissue consisting of an internal part (bone, cartilage, blood vessels) and an external part, the epidermis. The latter, whether from wild or domestic deer, is in contact with the environment, interacting with dust, plants, pathogens carried by the wind or even faeces (particularly in farm animals), in addition to the bacterial and fungi flora that normally live commensally on the skin without causing pathology. All these are sources of infectious outbreaks by microorganisms in the worst case (Palmer et al., 2019), but even if they are not pathogenic, they can ruin laboratory experiments carried out on cell cultures.

The exposure of the antler tip to the environment is an important factor when considering the level of contamination. This tissue is more exposed to everything that encounters the deer than the base, which is more protected. Hyun et al. (2023), point out that after subjecting an environmental sample to high temperatures, such as autoclaving or pasteurization, certain pathogens can remain active for up to 60 days in high humidity storage Haga clic o pulse aquí para escribir texto. In this sense, freeze-drying as a preservation method would also affect the survival of microorganisms, since if we compare lyophilised and non-lyophilised DVA-T, we see that the latter has contaminants after the treatments that are not present when lyophilised (Ghorbani et al., 2008). Once the samples have been sterilised, it is necessary to know the time and conditions of storage so as not to encourage microbial repopulation (Trevors, 1996). Although UV radiation is one of the most widely used methods in the medical field for sterilising clinical equipment, it does not reduce the total number of microorganisms present after the samples have been treated. Articles show that this type of wave is able to eliminate from 70% to 90% of the amount of microorganisms, but the remaining microbiota are able to survive depending on the storage method; in this sense, sterilisation by UV radiation can be considered as a complementary procedure (Ploydaeng et al., 2021).

Sterilisation by filtration with PES membranes has achieved a 95% confidence level in other studies, with traces of contaminants able to repopulate the area (Colon et al., 2023). However, the effectiveness in removing microorganisms depends not only on the type of membrane but also on the manufacturer, as different materials used by companies have been shown to achieve different performance rates, which is largely related to product selectivity (Casino et al., 2023). In this case, the membranes used come from the same manufacturer and, unlike the CA, RC and MCE membranes, the PES membrane is considered to be the best option because, thanks to its conformation, it 1) does not allow the passage of microorganisms, 2) does not alter the structure of the proteins present and 3) does not affect the effectiveness of the extract in exerting anticancer activity, since PES membranes have fewer hydrogen bonds and do not interact with ionic forces capable of retaining and denaturing proteins (Sun et al., 2023). This is because they are polymers that can be used in biomedical applications thanks to their stable characteristics with respect to changes in the chemical environment, do not generate reactivity with proteins and do not produce apparent cytotoxicity in the human organism (Khan et al., 2023). Similarly, CA, RC and MCE membranes have a low filtration flux, equivalent to 15%–30% less than a PES membrane (Hwang and Chiang, 2014).

DVA has previously been shown to have direct anticancer activity on several tumour types, including prostate, glioblastoma, breast, colon and leukaemia (e.g., Yang et al., 2017; Chonco et al., 2021; Cao et al., 2023; Rosetti et al., 2024). In these studies, DVA was shown to reduce cell proliferation and migration. Several factors present in the extract may influence these results. A very important family of these factors are proteins. The study by Hu et al. (2015) and Cao et al. (2023) shows the anticancer effect of DVA based on only a monomeric peptide on breast cancer cells (Hu et al., 2015), or sarcoma 180 (Cao et al., 2023). Therefore, taking extreme care of proteins with a bioactive effect is essential and their denaturation would mean the loss of their potential anticancer action. In this respect, the extract profile showed that all methods, except for digestion and autoclaving, largely preserved the proteins and their molecular characteristics (Figures 6, 9). In digestion, the use of proteinase and acid pH favours the degradation of some of the protein content into peptides. The degradation of a population of proteins renders them undetectable for the BCA colorimetric technique (Figure 6). The BCA reaction focuses on the biocinchoninic acid and Cu salt complex+1, which is able to perform atom transfer with the peptides in the sample with greater sensitivity than other techniques and high stability (Smith et al., 1985). However, the sensitivity limit of the method is at microgram protein concentrations, with small peptides and amino acids being undetectable (Betti et al., 2021). Figures 6, 9 show only detectable protein and peptide concentrations. It is important to note that the presence of detectable concentrations does not guarantee that these molecules exert significant biological effects in cells. Furthermore, proteins that are not detectable in these analyses should not be dismissed as having no biological relevance. This can be seen from the effect they exert on contact with tumour cells, where in a pasteurized sample they rapidly show an effect on SW480 and SW620 cells (Figure 10). This is not the only case, as some studies showed strong anticancer effects even when the DVA extract was supplied orally and therefore it was digested (e.g., Rossetti et al., 2024).

On the other hand, exposing a set of proteins to high temperatures and pressures causes aggregation and degradation, which occurs in autoclaves (Messens et al., 1997). Thus, Figure 9 shows that the sample does not pass through the gel and is retained when loaded. Agglutination corresponds to a change in the hydrophobic structure of the molecule and its activity due to the high pressure over a given time, which causes an increase in hydrogen bonds (Grigera and McCarthy, 2010). When this happens, the protein unfolds, loses its three dimensional structure and loses its activity as it can no longer be taken up or cross the cell membrane. Indeed, the autoclaved extract fails to visualize in the gel, this may be due to the degradation process, which is perhaps more likely. Although in addition to degradation, it could also be attributed to the formation of large protein aggregates during autoclaving, which could hinder their migration through the gel. However, in a denaturing gel such as the one used, these aggregates would denature in their quaternary structure, so it is more unlikely to be the original reason, although this hypothesis cannot be totally ruled out.

After evaluating the antitumour effect of the different extracts on colorectal cancer (SW480 and SW620) cell lines, the results obtained provide a detailed insight into how the processing and sterilisation methods affect the cell viability and efficacy of the different extracts (lyophilised and non-lyophilised DVA-T and DVA-B). For the primary SW480 tumour cell line, untreated and lyophilised extracts showed a significant reduction in cell viability with the DVA-T and DVA-B extracts. This suggests that lyophilisation without further treatment may be sufficient to maintain the antitumour properties of these extracts, especially in the antler tip, where the most pronounced effect is observed (37% ± 10%). On the other hand, in the non-lyophilised extracts, it was observed that most of the DVA-B samples showed a significant decrease in cell viability. Nevertheless, DVA-T filtered with PES membrane was the only condition that showed a significant antitumour effect, lower than DVA-B. In contrast, lyophilised extracts of DVA-B did not show significant antitumour effects in the SW620 cell line derived from metastases of the same patient. However, rapid pasteurization proved effective, reducing cell viability by up to 57% ± 5%. Both lyophilised and non-lyophilised DVA-T extracts showed antitumour efficacy when filtered with PES membrane. The improvement in cell survival in SW620 line for DVA-T is due to metastatic changes and expression of enzymes capable of modulating substrate passage such as CD26, positive regulation of epithelial-mesenchymal transition and TGFβ signatures, while no differences were seen in the response of SW480 and SW620 when exposed to DVA-B.

It should be noted that some limitations could affect the results of this study making it different if they are tried to be replicated: 1) the speed of growth of the antler varies along the antler cycle and, if the anticancer properties are a response to the risk of developing cancer related to this speed, then different levels of anticancer effect could be expected depending on the stage of antler growth, age of animal, and even interindividual differences in antler extract composition; 2) the anticancer effect also differs between tumour type (e.g., glioblastoma, breast) and cell line (primary, secondary, resistant or not); 3) there is the possibility that the results may be different if anticancer compounds would be extracted with solvents different to water. Therefore, the final product is heterogeneous, with non-standardized protein production. In this sense, in the future it would be desirable to identify the important active principles in the processes described and their purification and combination for the study free of excipients and possible inhibitory or antagonistic molecules of the processes studied.

Those anticancer effects are due to different conditions are described in the literature. On the one hand, there is the area of the antler that is worked on: Rossetti et al. (2024) show reduced viability of breast and colorectal cancer cells after incubation with extracts from the tip and middle section of the antler, achieving higher anticancer activity with the tip. Similarly, Chonco et al. (2021) when testing the extract on glioblastoma cells, obtained greater anticancer activity in the tip than in the middle section of the antler. On the other hand, a second liophilisation conducted on DVA powder renders affects certain bioactive compounds, rendering them inactive on certain cell types. Among the various factors that promote protein denaturation are low temperatures before to the liophilisation in the frozen sample, which deactivate enzymes, causes loss of physical properties due to increased solute concentration, surface denaturation at the ice water interface, pH changes during freezing, and removal of the protein hydration layer (Cubas et al., 2009). Finally, the type of sterilisation used will affect the bioavailability of the protein products and other compounds that exert the anticancer effect, which may be different for each type of tumour, which would explain why certain treatments work better in primary colorectal cancer.

To assess the influence of lyophilisation on the bioactivity of the extract, the expression of biomarkers of tumour resistance and progression was evaluated in the SW480 colon cancer cell line. The results show significant changes in the expression of several key biomarkers of tumour resistance and progression. The data obtained suggest that lyophilised DVA activates resistance pathways in SW480 tumour cells. Furthermore, lyophilised DVA-T extract induced an additional overexpression of RAGE (84.1% ± 0.6%), which may indicate a specific modulation of inflammatory pathways and tumour progression (Wang et al., 2015).

On the other hand, non-lyophilised extracts showed a different biomarker expression profile. The non-lyophilised DVA-T extract increased the expression of TROP2 (87.6% ± 0.6%), a marker associated with tumour aggressiveness (Cubas et al., 2009; Kuai et al., 2020). However, non-lyophilised DVA-B extract decreased the expression of TROP2 (42% ± 10%) suggesting a possible inhibitory effect on tumour proliferation and resistance (Fang et al., 2009; Sánchez-Díez et al., 2022). In addition, the non-lyophilised DVA-T extract reduced the expression of AC133 (79% ± 10%), a cancer stem cell marker, which could imply a reduction in the self-renewal and resistance capacity of tumour cells, however, it does not achieve significant differences (Jaksch et al., 2008; Aguirre-Ghiso, 2021). Nevertheless, the significant differences between the effect of lyophilised and non-lyophilised DVA extracts on colorectal cancer protein expression may be due to the different preservation of the protein structure in both cases. Noteworthy, in all cases of significant differences between lyophilised and non-lyophilised DVA extracts, the increase in protein expression is greater with lyophilised DVA, which may be due to the mentioned preservation of the protein structure and related to its greater antitumour capacity.

These observations are consistent with recent studies indicating that sterilization approaches involving high temperature (Tao et al., 2021; Garcia-Castello et al., 2024; Siddiqui et al., 2024) and pressure can significantly alter protein conformation and reduce measurable biological activity, whereas membrane-based filtration methods tend to preserve molecular stability and functional integrity. Furthermore, contemporary methodological reviews emphasize that protocol optimization is critical when working with complex natural extracts, as minor variations in sterilization or storage conditions may substantially influence reproducibility and downstream *in vitro* outcomes (Evrova et al., 2019; Postružnik et al., 2024). In this context, our findings align with current methodological recommendations that prioritize low-impact sterilization strategies to maintain extract stability while ensuring microbial safety.

As the SW480 cell line is derived from a primary colon cancer, there may be a subpopulation of non-tumour quiescent cells expressing the Lgr5+ marker, as presented by some authors and shown in Figure 11 (Hirsch et al., 2014; Planutis et al., 2015). Deer antler, known for its regenerative capacity in normal stem cells, may act in a similar way on this Lgr5+ population, promoting their differentiation and regeneration (Li, 2023). This effect may be mediated by an increase in the expression of TROP2, a marker associated with intracellular calcium signalling and cell differentiation. The expression of hTROP-2 in response to antler extract treatment could indicate a calcium signalling mechanism that favours cell regeneration, similar to the behaviour observed in normal stem cells, suggesting a potential therapeutic application of antler components in the modulation of quiescent stem cells in tumour and non-tumour contexts (Omori et al., 2022). Similarly, the SW480 line, derived from a Duke B patient and a small tumour, has a high potential for cellular heterogeneity and therefore contains several subpopulations, including a CD3^+^ subpopulation previously reported by Wang et al. (2022). This is a critical marker in the T cell receptor complex and the activation of immune interactions, so its overexpression would enhance the immune response in the body.

Overall, our study demonstrates that preserving protein integrity is essential when handling bioactive extracts, as denaturation may result in the loss of measurable *in vitro* biological activity. In this respect, the extract profile showed that most sterilization methods, with the exception of digestion and autoclaving, largely preserved protein composition and molecular characteristics. However, it should be noted that these findings are applicable to DVA obtained using a highly polar solvent such as water, which primarily concentrates hydrophilic biomolecules. Ongoing studies in our laboratory are evaluating the extraction capacity of alternative solvents. Collectively, our results highlight the importance of methodological optimization and standardized processing conditions to maintain extract stability and reproducibility for subsequent biological evaluation.

## Data Availability

The raw data supporting the conclusions of this article will be made available by the authors, without undue reservation.
