# Supplementary material for: Comparison of sterilisation methods for deer velvet antler extracts and their impact on anticancer activity
Source: Front Pharmacol. 2026 Apr 20;17:1782990. doi: 10.3389/fphar.2026.1782990 (PMC13136180; doi:10.3389/fphar.2026.1782990)
Supplement: Supplementary file 1 [file Supplementaryfile1.pdf]

Supplementary Material 1| Equipment, reagents, materials, gastric simulation buffer components, antibodies, and programs used in the research.

Equipment	Model	Supplier	Country
Mechanical saw	PHKSA 20 A2	Parkside	Sajonia, Germany
Ultra-low temperature freezers	NU-9668E	NUAIRE	Plymouth, Minnesota, USA
Sublimator	30EKS	ZIRBUS technology GmbH	Bad Grund, Germany
Blade mill	Retsch SM300	Retsch GmbH	Haan, Germany
Mixer mill	Retsch MM400	Retsch GmbH	Haan, Germany
Milli-Q water dispenser	Ultramatic GR	Wasserlab	Barbatáin Noáin, Navarra, Spain
Magnetic stirrer	SBS-MR-1600/6	Steinberg systems	Hamburg, Germany
Microcentrifuge	UNIVERSAL 320	Hettich	Tuttlingen, Germany
Freeze-dryer	BIOBASE BK-FD10PT	BIOBASE BIODUSTY	Wolfenbüttel, Germany
Thermal block	Hb120-S	Westtune	Hangzhou, Zhejiang, China
Laminar flow cabinet	AireGard ES, NU-126 series	NuAire	Plymouth, Minnesota, USA
Autoclave	BAVT-102	Biolab	Barcelona, Spain
Benchtop pH meter	PHS-W	Bante Instrument	Shanghai, China

Orbital shaker incubator	LT-X (Lab-Therm)	Adolf Kühner	Birsfelden, Basel, Switzerland
Microplate reader	BIOBASE-EL 10th	BIOBASE BIODUSTY	Wolfenbüttel, Germany
CO_2 Incubator	3111, Series II water Jacker	Thermo Fisher Scientific	Marietta, Ohio, USA
Microscopy	Motic AE2000	MoticEurope	Barcelona, Spain
Basic Power Supply	PowerPac™	Biorad	Hercules, California, USA
Microplate shaker	PMS-1000i	GRANT bio	Cambridge, UK
Imaging system	ImageQuant LAS 500	GE Healthcare Life Sciences	Chicago, Illinois, USA
Cytometer	FACS Canto cytometer	Becton Dickinson	Franklin Lakes, Nueva Jersey, USA

Reagent	**Code**	**Supplier**	**Country**
Xylazine	26200022	Laboratory Calier	Barcelona, Spain
Ketamine	2529ESP	Merial	Lyon, French
PBS 10X	SH30258.01	Cytiva	Burlington, Massachusetts, USA
DMEM medium	10-017-CV	Dominique Dutscher	Bernolsheim, France
Fetal Bovine Serum	P30-3302	Pan BioTech	Aidenbach, Germany
L-Glutamine	P04-82050	Pan BioTech	Aidenbach, Germany
LB Lennox	LBBR-00P-500	Labbox Labware S.L.	Barcelona, Spain

BCA Protein Assay Kit	71285-M	MerckMillipore	Massachusetts, USA
Prestained Protein Ladder	ab116028	abcam	Cambridge, UK
SureCast™ Gel Manual Preparation Pack A	15583371	Thermo Fisher Scientific	Waltham, Massachusetts, USA
novex MES-SDS buffer	13266499	Life Technologies	California, USA
Coomasie blue G-250 5 mg	1.15444	Sigma Aldrich	Burlington, Massachusetts, USA
Penicillin/streptomycin	30-001-CI	Corning	New York, NY, USA
Thiazolyl Blue Tetrazolium Bromide (MTT)	A2231	BioChem, PanreacApplichem	Barcelona, Spain
DMSO	DMSO-00A-1K0	Labbox Labware S.L.	Barcelona, Spain
BSA	A9418	Sigma Aldrich	Burlington, Massachusetts, USA
Glyo-Fixx™	9990924	Thermo Fisher Scientific	Waltham, Massachusetts, USA
Materials	**Code**	**Supplier**	**Country**
Filter with Polyethersulfone (PES) membrane of 0.22 µm	SFPE-12E-100	Branchia	Barcelona, Spain
Filter with Cellulose acetate (CA) membrane of 0.22 µm	SFCA-12E-100	Branchia	Barcelona, Spain
Filter with mixed cellulose esters (MCE) membrane of 0.22 µm	SFMC-122-100	Branchia	Barcelona, Spain

Filter with RC membrane of 0.22 µm	729236RC	BioChem, PanreacApplichem	Monza, Italy
96-well plates Non steril	390968	BIOSIGMA	Barcelona, Spain
96-well plates Steril	390965	BIOSIGMA	Barcelona, Spain
12-well plates	D200002	Deltalab S.L	Barcelona, Spain
Reagent	**Code**	**Supplier**	**Country**
Potassium chloride	POCL-00A-1K0	Labbox Labware S.L.	Barcelona, Spain
Monopotassium phosphate	PODP-00A-500	Labbox Labware S.L.	Barcelona, Spain
Sodium hydrogen carbonate	27780.291	VWR International	Pennsylvania, USA
Sodium chloride	131659	BioChem, PanreacApplichem	Monza, Italy
Magnesium chloride hexahydrate	131396	BioChem, PanreacApplichem	Monza, Italy
Ammonium carbonate	141116	BioChem, PanreacApplichem	Monza, Italy
Calcium chloride dihydrate	131232	BioChem, PanreacApplichem	Monza, Italy
Pepsin	9001-75-6	VWR International	Pennsylvania, USA
Antibody	**Code**	**Supplier**	**Country**
Anti-BCRP1	130-104-958	Miltenyi Company	Bergisch Gladbach, Germany
Anti-CD133	130-113-106	Miltenyi Company	Bergisch Gladbach, Germany

This is a provisional file, not the final typeset article

Anti-AC133	130-113-186	Miltenyi Company	Bergisch Gladbach, Germany
Anti-EPCAM	130-111-000	Miltenyi Company	Bergisch Gladbach, Germany
Anti-CD34	130-113-179	Miltenyi Company	Bergisch Gladbach, Germany
Anti-CD36	130-110-740	Miltenyi Company	Bergisch Gladbach, Germany
Anti-CD44V6	130-111-238	Miltenyi Company	Bergisch Gladbach, Germany
Anti-TROP2	130-115-055	Miltenyi Company	Bergisch Gladbach, Germany
Anti-CD44	130-114-535	Miltenyi Company	Bergisch Gladbach, Germany
Anti-DCLK1	ab202755	abcam	Cambridge, UK
Anti-RAGE	Ab237363	abcam	Cambridge, UK
Anti-LGR5	562912	Becton Dickinson (BD)	Franklin Lakes, Nueva Jersey, USA
Anti-CD166	559263	Becton Dickinson (BD)	Franklin Lakes, Nueva Jersey, USA
Program	**Version**	**Supplier**	**Country**
ImageJ	1.46j	National Institutes of Health (NIH)	Bethesda, Maryland, USA
GraphPad Prism	8.0.1	GraphPad Software Inc.	San Diego, CA, USA
FlowJo	10.8.1	Becton Dickinson (BD)	Franklin Lakes, Nueva Jersey, USA

Representative control plots

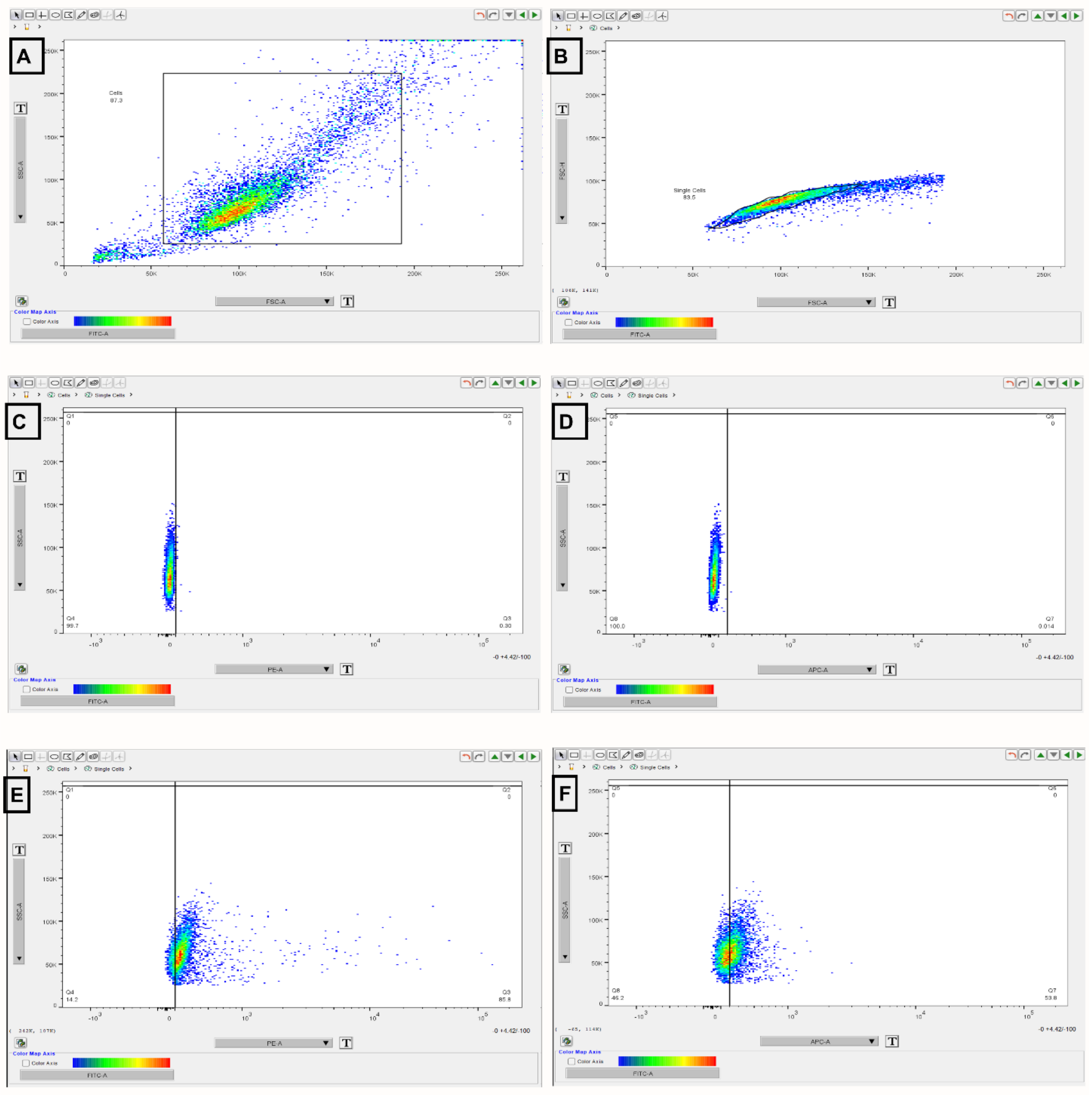

Supplementary material 2| Flow cytometry gating strategy and fluorescence controls. (A) Representative FSC vs SSC dot plot used to identify the main cell population and exclude debris. (B) FSC-A versus FSC-H plot showing the gating strategy applied to select single cells and exclude doublets. (C) dotplot showing the PE-negative population used to establish the background fluorescence threshold. (D) dotplot showing the APC-negative population used to define baseline fluorescence levels. (E) Representative dotplot displaying the PE-positive population after staining. (F) Representative dotplot displaying the APC-positive population after staining.
